# Selections

**Published:** 1892-02

**Authors:** 


### Selections.

Blaud's Pill in Anæmia.—"In his interesting articles on anæmia, Dr. Stephen Mackenzie discusses with some care and anxiety the proportions of alkali to be combined with sulphate of iron in Blaud's pill. Let me assure Dr. Mackenzie and your readers that these proportions are of no importance whatever, and that the alkali may be omitted without therapeutical loss, and with much practical convenience.

"For the last five years of my practice I ceased entirely to use the alkali, and my results were equally good. The mistakes and failures in treating adolescent and chlorotic anæmias are often due to the prevailing economy in the use of the iron. With five or even ten grain doses of citrate of iron little real progress may be made in many cases. No form of iron is so efficient as the sulphate, of which gr. j. thrice daily is to be given for a week, then two grain doses for ten days, and so on till nine, or or even twelve grains are taken in the day. The drug should be gradually reduced in like manner, and the course should never be less than three months in duration, or relapses may occur. In obstinate cases, the addition of one-thirtieth grain of strychnine, or one-quarter grain of phosphide of zinc are invaluable aids. Most patients require the inclusion of one-third to one-fourth grain of extract of aloes to prevent the constipating effect of the sulphate, but Dr. Mackenzie rightly denies that constipation is the cause of chlorosis, or even generally coincident with it. This error is due to reasoning from an insufficient number of careful records.

"Iron pills should be carefully made from the dried sulphate, and not with gums, which, by hardening, make the pills insoluble. In any case it is better to order the pills to be freshly made every week, if not even more frequently. Patients who are unable to take pills are best treated with the saccharated carbonate of iron, of which three or four large teaspoonfuls may be given in the day."—*Dr. T. C. Allbutt, in Brit. Med. Journal.*

DIPHTHERIA.—I should like to put on record a method which I have found exceedingly useful in the treatment of diphtheria ; it consists in the hourly spraying of the fauces and naso-pharynx with a saturated solution of biborate and bicarbonate of soda (about forty grains of each to the ounce of water). The *rationale* of the thing is, I believe, that the bicarbonate of soda tends to loosen and liquefy the tenacious mucus which is always present in these cases, and thus allow the borax to exert its action as an antiseptic ; it is of course a weak antiseptic, but it has the advantage of being compatible with the bicarbonate, which I believe plays an important part as I have said above.

I have not the smallest doubt, in my own mind, that it tends to shorten the course of the disease; in fact, in one case the recovery was so rapid that I should have doubted my diagnosis if it had not been that a sister of the patient, who was living in the same house, had just died of the disease in St. George's Hospital.

In severe cases it is well to spray up the nostril to prevent the membrane spreading upwards, but for this the solution should be diluted, for if it is saturated with the alkali it causes hyperemia and soreness of the mucous membrane.

In justice to Dr. Fenwick, I must say that in all my cases I have given iron.

It is now nearly two years since Dr. Coall mentioned the line of treatment to me; he tells me that he had used it for several years previously—in fact, it is to him that the credit is altogether due if the method should prove useful after a more extended trial.—*Vernon Jones, M. D., in British Medical Journal.*

---

THE ÆTIOLOGY OF DIPHTHERIA.—In the *Johns Hopkins Bulletin*, Dr. W. H. Welch gives the latest results of the researches on this point and adds from his own studies still other observations. From all this he concludes it is fully proved that the specific primary cause of diphtheria is the Klebs-Loffler bacillus. This organism he calls the *bacillus diphtheriæ.* This bacillus is present in every case of primary diphtheria in such number and situation as to explain the local manifestations of the disease. It can be isolated in pure culture readily, and a disease identical in all respects with human diphtheria can be produced experimentally by the inoculation of pure cultures. Thus we are in possession of positive means for making a diagnosis of diphtheria. The method of doing this is not difficult and can readily be applied, though it may be questioned whether many practitioners are likely to make use of these means. We are taught that there are pseudo-membranous anginas which must be separated from etiologically pure diphtheria, and that diphtheria may exist in extremely mild forms even without visible pseudo-membranous deposits. The endless controversy as to whether diphtheria is primarily a local or general disease is settled. It is primarily local, the grave constitutional symptoms are the result of intoxication with poisonous products formed by the local action of the bacilli. We can study the varied effects produced upon the animal body by the specific toxic products of the diphtheritic germ. We can separate the alterations belonging to the disease itself from the many complications of the disease. Intelligent measures of prophylaxis can be based upon a definite knowledge of the characters of the specific germ and its behavior in the body. Rational indications for treatment can be established, and have been formulated.—*American Lancet.*

---

PROSTITUTION.—In Vienna each prostitute receives a book containing a description and photograph of herself, and a copy of the laws relating to prostitution. No one under sixteen can be registered, nor persons afflicted with organic or constitutional disease. Sanitary examinations are made twice a week, all diseased women are put into hospitals, primary syphilitic cases are quarantined for three months, and kept under treatment for two years. Clandestine prostitutes are treated in the same way by their own physicians.

In France, M. Commenge recently stated, at a meeting of the Academy of Medicine of Paris, that he had collected the statistics of the number of diseased prostitutes found in the decade from 1878 to 1887: First, among women registered by houses or cards; second, among those women that—though registered—

were the objects of more or less frequent arrests, and constituted a special class under the name of *femmes du depot;* third and lastly, among the uninspected, or women that lived by clandestine prostitution. The result obtained, based on nearly a million visits, showed the number of cases of syphilis in each thousand examined to be respectively 3.1, 2.7 and 23.9. Of other venereal diseases 3.0, 2.4 and 14.5.

It is only by the accumulation of such statistics that the fanatical sentiment against the regulation of prostitution can be overcome and the health of innocent women and children protected.—*Boston Med. and Surg. Jour.*

---

PLACENTA PRÆVIA.—Braxton Hicks lays down the following rules as regards treatment:

1. The diagnosis of placenta prævia once established you must terminate labor with the least possible delay.

2. When the operation has been commenced you must not leave your patient until it is completed.

3. When the cervix is completely dilated and the placenta is marginal, it is necessary to break the membranes, and see whether the head is pushed toward the neck during the pains.

4. If the head descends slowly, employ the forceps or perform version.

5. If the os is small and is more or less covered by the placenta, the latter should be cautiously detached around the whole circumference of the os. If there is no hæmorrhage delay of one or two hours is permissible. If the os is not dilated and if you have no dilator at hand, you may dilate the cervix manually. If you think that the forceps may be easily applied, it is well to apply them. If not it is necessary to do bi-polar version by the combined internal and external method. The cervex is thus tamponned by the legs or breach of the child. After this the treatment is the same as for any case of foot or breach presentation.

6. If the neck is small, and if you have neither forceps nor dilator, version must be done.

7. If during the manœuvers here mentioned, a violent hæmorrhage occurs version must be terminated by extraction of the fœtus.

8. When the fœtus is dead, or when labor comes on before the end of the seventh month, it is necessary to do version and leave the rest to nature.—*L' Union Med.*—*West Med. Review.*

---

THE SPEEDY CURE OF TONSILLITIS.---The *London Medical Recorder* contains the following prescription for the rapid relief of tonsillitis:

℞ Tinc. of veratrum viride, 30 minims.
Sulphate of morphia, 1½ grains.
Distilled water, 6 drachms.

M.—Dose, one teaspoonful, given twice, with one hour's interval at the outset of the treatment, and then at intervals of two or three hours, as may be required.

The author of the treatment holds that there is some kind of therapeutical agreement or harmony between the drugs, when used together, which gives them an efficiency not possessed by either of them when used separately. For example, the liability to nausea from either of them alone is greatly modified by the combination. He refers to a number of cases in which this treatment has seemed to produce unusually prompt relief, and he asserts that he knows of no drug or drugs which have the power to control tonsillar inflammation with the certainty and celerity of those agents when used jointly.—*Ex.*

---

FRACTURE OF THE SHAFT OF THE FEMUR.—At the last meeting of the American Surgical Association a committee, consisting of Dr. Stephen Smith, of New York; Dr. Agnew, of Philadelphia ; Dr. Parkes, of Chicago ; Dr. Cheever, of Boston ; Dr. McGuire, of Richmond, and others, made a report on "What should be considered satisfactory results in the treatment off racture of the shaft of the femur ?" Following is a summary of their report :

*Conclusions.*—A satisfactory result has been obtained in the treatment of fracture of the shaft of the femur when—

1. Firm bony union exists.
2. The long axis of the lower fragment is either directly continuous with that of the upper fragment or the axes are on nearly parallel lines, thus preventing angular deformity.
3. The anterior surface of the lower fragment maintains nearly its normal relation to the plane of the upper fragment, thus preventing undue deviation of the foot from its normal position.
4. The length of the limb is either exactly equal to that of its fellow, or the degree of shortening falls within the limits found to exist in 90 per cent. of healthy limbs, viz., from one-eighth of an inch to one inch.
5. Lameness, if present, is not due to more than one inch of shortening.
6. The conditions attending the treatment prevent other results and those obtained.—*Medical News.*

---

RECENT RECIPES FOR GONORRHŒA—Brindisi (*Rev. Gen. de Therap.*) employs the following anti-septic injection ;

℞. Antipyrin........................gr. xlv.
Sulphate of zinc..................gr. iv.
Rose water.
Cherry-laurel water..............aa ℥ij. Mix.

Dr. William B. Dewees, of Salina, Kan., says (*Kan. Med. Jour.*) few cases will remain uncured after eight days' use of injection of

℞. Sodium biborat.

Resorcin..........................aa ʒss.

Glycerin..............................℥iiss.

Rose water, q. s.........................℥viij.

M. S.—Inject ʒij every two hours the first day; then lengthen intervals as the discharge lessens. After third day, take internally, tincture Canabis indica, 5 drops every three hours. Bathe glans penis in as hot water as can be borne three times daily.

Dr. Richard Lee (*Intern. Surg. Jour.*, August, 1891) first uses warm injections of sodium biborate and morphia [sulph.] (in glycerin and rose water) for three days; and then *aristol* in liquid vaseline—25 grains to ounce. Prompt relief without relapse was effected in from four to six days.—*Virginia Medical Monthly.*

---

TREATMENT OF EPILEPSY.—Under this head Poulet, in *Bulletin Generale de Therapeutique*, writes of a combination of bromide potassium with calabar bean, which has given him success in the treatment of obstinate cases of epilepsy where the bromides alone had failed. A favorite formula of his is :

| | | |
|---|---|---|
| ℞. | Bromide of potassium, . . . | 100 parts. |
| | Tincture of calabar bean, . . | 35 " |
| | Water, . . . . . . . . . . | 470 " |

Sig: A tablespoonful, to be increased to a tablespoonful and a half, then two tablespoonfuls, daily.

A tablespoonful contains about 57 grains of bromide, and about 16 minims of the tincture. The medicine may be given in divided doses instead of in one full dose, half a teaspoonful being given at first twice, then three times, then four times a day.

Poulet reports five obstinate cases treated in this manner. These were cases where bromide alone failed to cure.

Poulet terminates his article by the following conclusions:

The bromides remain the sheet anchor in the treatment of epilepsy—and by the term "bromides" we have especial reference to the bromide of potassium, which alone is truly efficacious.

There are, however, a great many epileptics whose attacks are only mitigated or postponed, not completely suppressed, by bromide of potassium.—*Times and Register.*

---

THERE are 2,500 licensed physicians in New Jersey, and of these there are said to be ten per cent. registered on bogus or fraudulent diplomas.

BRONCHIAL ASTHMA.

℞. Iodine of ammonium, dr. ij.
Fl. ext. grindelia robusta, ℥ss.
Fl. ext. glycyrrhiza, dr. iv.
Tinct. lobelia,
Tinct. belladonna, aa dr. ij.
Syr. tolu, q. s. ad. ℥iv.

Dose.—Teaspoonful three times a day; extra doses during a paroxysm.

This formula may be varied to suit indication. I have cured many cases of asthma by means of it, some of over twenty years' standing, myself among the number.—*Dr. Covert in American Medical Journal.*

---

ROUGH AND READY TEST FOR CANCER.—Mr. H. J. Stites, of Edinburgh, proposes the following as a quick method of diagnosis for carcinoma at the operating table: 1. Excise the mamma. 2. Wash thoroughly in water to remove blood. 3. Place in a five per cent. solution of nitric acid for ten minutes. 4. Wash in cold water for five minutes. By the time these procedures are executed the axilla is cleaned out and the vessels tied. The mamma is now examined. The carcinomatous structure appears a dull white, like the eye of a boiled fish; the healthy tissue translucent. When such reaction is seen, additional tissue should be removed at the corresponding point.—*Journal American Medical Association.*

---

DIED.—In Augusta, Ga., December 15, ult., Dr. Henry T. Campbell, aged sixty-eight. The Georgia profession has sustained a great loss in the death of this excellent physician and estimable gentleman. Dr. Campbell was once Professor of Anatomy and Surgery in the New Orleans Medical College and in 1885 was president of the American Medical Association. At the time of his death he was Professor of Surgery and Gynecology in the Augusta Medical School. In spite of a large practice he made frequent and valuable contributions to medical literature.

### THE INTERNATIONAL EXECUTIVE COMMITTEE OF THE PAN-AMERICAN MEDICAL CONGRESS.

The Committee on Organization of the Pan-American Medical Congress at its meeting at St. Louis last October elected the following International Executive Committee: The Argentine Republic, Dr. Pedro Lagleyze, Buenos Ayres; Bolivia, Dr. Emelio Di Fomassi, LaPaz; Brazil, Dr. Carlos Costa, Rio de Janeiro; British North America, Dr. Jas. F. W. Ross, Toronto; British West Indies, Dr. James A. DeWolf, Port of Spain; Chili, Dr. Moises Amaral, Santiago; United States of Columbia, Dr. P. M. Ibanez, Bogota; Costa Rica, Dr. Daniel Nunez, San Josè; Ecuador, Dr. Ricardo Cucalon, Guayaquil; Guatemala, Dr. Josè Monteris, Guatemala Nueva; Hayti, Dr. D. Lamothe, Port au Prince; Spanish Honduras, Dr. George Bernhardt, Feguagalp; Mexico, Dr. Fomas Noriega, City of Mexico; Nicaragua, Dr. J. I. Urtecho, Grenada; Peru, Dr. J. Casamira Ulloa, Lima; Salvador, Dr. David J. Guzman, San Salvador; Spanish West Indies, Dr. Juan Santos Fernandez, Habana; United States, Dr. A. Vanderveer, Albany, N. Y.; Uruguay, Dr. Jacinto DeLeon, Montevideo; Venezuela, Dr. Elias Rodenguez, Caracas.

Hiwaii, Paraguay, Santo Domingo, the Danish, Dutch and French West Indies are not yet organized. Nominations of local officers have been received from a majority of all the members of the International Executive Committee and a number of the lists have been confirmed by the Committee on Organization. These will be announced as rapidly as acceptances are examined.

CHARLES A. L. READ,
Secretary-General.

Cincinnati, January 15, 1892.

---

REMEMBER IN PRESCRIBING IRON.—That it has never been proved that salts of iron are absorbed in the intestinal canal.

That there is considerable evidence to the contrary.

That however minute the dose of iron administered, the stools are invariably blackened by reduction of the iron to the state of sulphide, which is insoluble in the alkaline of neutral intestinal juices.

That there is a very general opinion in favor of the old-fashioned perchloride of iron in cases where iron is indicated.

That this opinion is undoubtedly proven by long experience, and that no other inorganic preparation of iron gives better results *where tolerated.*

That the effect of perchloride of iron is *not* proved to depend on the iron at all, and that it is probably due chiefly to the mineral acids, which are always present in tincture, or solution of perchloride of iron.

That a neutral solution of perchloride of iron is not permanent, and cannot be used in pharmacy.

That the neutral proto or perchloride, carbonate, sulphate, phosphate, and all other salts of iron, not excepting the so-called albuminates, peptonates, and dialyzed preparations of iron, cannot be relied on to give as good results as the perchloride.

That Sir Andrew Clark has long held that constipation, when complicated with anæmia, is due to the decomposition of food (and with it the iron compounds), with absorption of ptomaines, which produce a continuous toxic effect and enfeeble the red blood corpuscles, besides depriving them of their natural source of iron, which is necessary to maintain their normal oxygen-bearing power.

That if iron be administered at all, the salts of iron should be rigorously avoided, as they are liable to intensify the anæmia, rather than do good, besides blackening the teeth, and frequently disagreeing with the patients.

That hemoglobine (Ferrum Sanguinis) is an ideal hæmatinic, it *does not blacken the stools* (thus proving its assimilation), and is the most prominent ferruginous element of our food in a very concentrated form.

That Ferrum Sanguinis is a dry, semi-crystalline powder, isolated from bullock's blood; this is soluble in water, but is best dispensed in small spherical capsules (Chapoteaut) of twenty centigrammes each, which is equivalent to one milligramme of metallic iron.—*St. Louis Medical and Surgical Journal.*

